# Retraction: Comparison of the Performance of Various Scores in Predicting Mortality Among Patients Hospitalized With COVID-19

**DOI:** 10.7759/cureus.r43

**Published:** 2022-03-17

**Authors:** Daniyal Jilanee, Shamshad Khan, Syed Muhammad Huzaifa Shah, Natalia M Avendaño Capriles, Camilo Andrés Avendaño Capriles, Hareem Tahir, Afreenish Gul, Syed U Ashraf, Sohaib Tousif, Ahsun Jiwani

**Affiliations:** 1 Medical College, Liaquat National Hospital and Medical College, Karachi, PAK; 2 Respiratory Medicine, Blackpool Victoria Hospital, Blackpool, GBR; 3 Medical Education, Ziauddin University, Karachi, PAK; 4 Medicine, Universidad del Norte, Barranquilla, COL; 5 Foundations of Clinical Research (FCR) Program, Harvard Medical School, Boston, USA; 6 Medical College, Army Medical College, Rawalpindi, PAK; 7 Internal Medicine, Ziauddin University, Karachi, PAK; 8 Internal Medicine, Dow International Medical College, Karachi, PAK; 9 Medicine, Ziauddin University, Karachi, PAK; 10 Indus Hospital Research Center, Indus Hospital and Health Network, Karachi, PAK

This article has been retracted due to the unknown origin of the data, lack of verified IRB approval, and purchased authorships. It was also discovered that the article was not submitted by Ahsun Jiwani, but by Rahil Barkat while using the account of Ahsun Jiwani. Mr. Barkat was involved in data theft and misuse in two recently published Cureus articles, which have since been retracted.

As the origin of this article’s data and verified IRB approval cannot be confirmed, we have made the decision to retract this article. Cureus has confirmed that the co-authors were asked by Mr. Barkat to proofread the article and provide payment in exchange for authorship. (Proofreading is an insufficient contribution to warrant authorship as defined by ICMJE.) These payments were made in the guise of “editing fees” but greatly exceed any editing fees paid to Cureus. While these authors may have been defrauded by Mr. Barkat, they remain complicit due to their lack of honest contributions to the article.

